# Long-Term and Carryover Effects of Supplementation with Whole Oilseeds on Methane Emission, Milk Production and Milk Fatty Acid Profile of Grazing Dairy Cows

**DOI:** 10.3390/ani11102978

**Published:** 2021-10-15

**Authors:** Camila Muñoz, Rodrigo Villalobos, Alejandra María Teresa Peralta, Rodrigo Morales, Natalie Louise Urrutia, Emilio Mauricio Ungerfeld

**Affiliations:** 1 Instituto de Investigaciones Agropecuarias, INIA Remehue, Osorno 5290000, Chile; aperaltaferrada@gmail.com (A.M.T.P.); rmorales@inia.cl (R.M.); natalie.urrutia@inia.cl (N.L.U.); 2 Facultad de Ciencias Veterinarias y Pecuarias, Universidad de Chile, Santiago 8820808, Chile; rvillalobos.seelmann@gmail.com; 3 Instituto de Investigaciones Agropecuarias, INIA Carillanca, Temuco 4880000, Chile; emilio.ungerfeld@inia.cl

**Keywords:** oilseed, cottonseed, methane, grazing, milk fatty acid profile, carryover, dairy cow

## Abstract

**Simple Summary:**

Dairy cow diets that include oils have shown potential to decrease methane emissions, which contribute to climate change. However, there is limited information on long-term interventions for animals in grazing systems. The aim of this study was to evaluate the effects of feeding oilseeds on the persistency of methane mitigation effects and milk production of dairy cows during a spring and summer grazing season. Carryover effects into autumn were studied. Eight weeks after beginning the feeding trial, cottonseed was more effective than linseed or rapeseed in decreasing methane per kilogram of ingested feed, but the mitigation effect did not persist when evaluated 11 weeks later. All oilseeds maintained milk production in spring, but in summer, milk yield was lower with cottonseed. There were no carryover effects of feeding oilseeds, once supplementation ended. Thus, adding oils to dairy cow diets through cottonseed supplementation had only a temporary effect on methane mitigation. This long-term study, conducted under grazing conditions, can help to assess how proposed interventions to mitigate methane can affect production and sustainability aspects of grazing dairy systems.

**Abstract:**

Research is ongoing to find nutritional methane (CH_4_) mitigation strategies with persistent effects that can be applied to grazing ruminants. Lipid addition to dairy cow diets has shown potential as means to decrease CH_4_ emissions. This study evaluated the effects of oilseeds on CH_4_ emission and production performance of grazing lactating dairy cows. Sixty Holstein Friesian cows grazing pasture were randomly allocated to 1 of 4 treatments (*n* = 15): supplemented with concentrate without oilseeds (CON), with whole cottonseed (CTS), rapeseed (RPS) or linseed (LNS). Oilseeds were supplemented during weeks 1–16 (spring period) and 17–22 (summer period), and the autumn period (wk 23–27) was used to evaluate treatment carryover effects. Cows fed CTS decreased CH_4_ yield by 14% compared to CON in spring, but these effects did not persist after 19 weeks of supplementation (summer). Compared to CON, RPS decreased milk yield and CTS increased milk fat concentration in both spring and summer. In summer, CTS also increased milk protein concentration but decreased milk yield, compared to CON. In spring, compared to CON, CTS decreased most milk medium-chain fatty acids (FA; 8:0, 12:0, 14:0 and 15:0) and increased stearic, linoleic and rumenic FA, and LNS increased CLA FA. There were no carry-over effects into the autumn period. In conclusion, supplementation of grazing dairy cows with whole oilseeds resulted in mild effects on methane emissions and animal performance. In particular, supplementing with CTS can decrease CH_4_ yield without affecting milk production, albeit with a mild and transient CH_4_ decrease effect. Long term studies conducted under grazing conditions are important to provide a comprehensive overview of how proposed nutritional CH_4_ mitigation strategies affect productivity, sustainability and consumer health aspects.

## 1. Introduction

Research is ongoing to find dietary strategies that decrease methane produced by ruminant enteric fermentation. An effective anti-methanogenic strategy for pastoral dairy systems must be feasible to implement in grazing conditions, have persistent methane-mitigating potential, and no negative effects on production.

Lipid addition to ruminant diets has been reported as means to effectively decrease methane (CH_4_) emissions in a moderate manner [1,2,3]. There are several modes of action by which lipids affect rumen fermentation. They provide dietary organic matter that is not fermented in the rumen and is not available for methane production [1]. Additionally, lipids in the rumen can inhibit hydrogen-producing bacteria and protozoa which provide methanogens with hydrogen for CH_4_ production, and they can also be directly toxic to methanogens [4]. In addition, to a lesser extent, unsaturated fatty acids (FA), when biohydrogenated in the rumen, offer an alternative hydrogen sink to methane [5]. The extent of the methane decreasing effect depends on the lipid source [4] and its form of presentation [6], with medium chain fatty acids (FA) being the most effective, followed by polyunsaturated FA and monounsaturated FA [4,7].

Depending on the rate of FA release from feeds and exposure to ruminal microorganisms, dietary lipid addition may also impair rumen fermentation decreasing dry matter (DM) intake and ruminal fiber digestion [4,8], and lipid inclusion levels in dairy cow diets are usually recommended up to no more than 6–7% of diet DM. One strategy to avoid the negative impacts of an altered fermentation is feeding oilseeds, as they have shown lesser effects on fermentation because of a slower oil release into the rumen [8].

Dietary inclusion of oilseeds in dairy cow diets through its effects on rumen fermentation can also affect milk fatty acid composition [9,10], and there is interest in shifting saturated milk FA towards unsaturated, milk FA as these are perceived as healthier for human consumption [11].

There is a lack of studies that conduct direct comparisons between oilseeds on methane emissions [4,9]. Our previous study comparing different oilseeds sources available in southern Chile revealed that whole cottonseed was the most potent oilseed in decreasing CH_4_ emissions [10], although in that study we had hypothesized that the more unsaturated lipid source (linseed) would have a greater effect mitigating CH_4_ than cottonseed or rapeseed, as the effect of FA on methane mitigation appear largely dependent on their level of saturation [4,12].

That study was conducted on dairy cows in a confinement system with TMR over a 4-week period of supplementation. Indeed, many CH_4_ mitigation studies have been conducted under confined intensive conditions, with few studies performed under grazing and more extensive set-ups. Yet, globally a vast proportion of livestock production and CH_4_ emissions takes place in pastoral grazing systems during much of the year [13]. As enteric CH_4_ is proportional to feed gross energy intake, CH_4_ emissions from, grazing systems with smaller animals can produce substantially less CH_4_ than larger dairy cows in confinement systems [14]. Pastoral systems vary in the time cows spend on pasture, yearly calving patterns to maximize grass intake, and level of supplemental feed offered, among other factors. Dietary oilseeds could be applied to grazing dairy cows though concentrate supplementation at milking, being a practical strategy to apply on a commercial farm and the inclusion of whole oilseeds may be easier to adopt by most local farmers, who lack the equipment and facilities to process oilseeds.

There is also a lack of long-term studies on the effects of some of the CH_4_ mitigation practices that are being investigated in confinement, under grazing conditions. This lack of knowledge about the long-term effects of CH_4_ mitigation strategies in grazing systems includes not only effects on CH_4_ emissions and animal performance, but also other aspects such as product quality, and animal reproduction and health. This study aimed to evaluate the effects of supplementation with three different whole oilseeds on CH_4_ emissions and animal performance of grazing dairy cows throughout a spring-summer-autumn grazing season. In addition, we hypothesized that supplementation of dietary oilseeds in grazing cows can have residual effects after discontinuing oilseeds supplementation.

## 2. Materials and Methods

The experiment was conducted over 27-weeks (September 2016 to April 2017) at Instituto de Investigaciones Agropecuarias, INIA Remehue (40°31′ S; 73°03′ W; 65 m above sea level) in Osorno, Chile.

### 2.1. Animals, Experimental Design and Diets

Sixty Holstein Friesian cows with an initial body mass of 511 ± 61.2 (mean ± SD) kg and 49.5 ± 12.1 days in milk were used in a randomized block design study. The animals were blocked by parity, days in milk and milk production, and were randomly assigned to one of four treatments.

During the entire study cows were maintained on swards sown 5 to 7 years prior, composed mainly by perennial ryegrass (*Lolium perenne*) and white clover (*Trifolium repens*) offered ad libitum and grazed separately by the different treatment groups. The four dietary treatments consisted of supplementation with different concentrates based on steam-rolled corn, rapeseed cake, a vitamin and mineral mixture, and oilseeds, in varying amounts (Table 1). The treatments were: no oilseeds (Control, CON), whole cottonseeds with lint (CTS), whole rapeseeds (RPS), or whole linseeds (LNS). All oilseeds were offered whole, without mechanical processing. In the spring (weeks 1 to 16 of the trial), concentrates were supplemented at 5.3, 4.8, 4.1 and 4.3 kg DM/d for CON, CTS, RPS and LNS, respectively, to provide cows with similar amounts of energy (60 ± 5.3 MJ ME/d) and crude protein (0.66 ± 0.08 kg/d). The concentrates containing oilseeds were formulated to supply 0.47 ± 0.04 kg/d of fatty acids, compared to 0.17 kg/day of the CON concentrate. In the summer (weeks 17 to 22 of the trial), the concentrate supplementation levels were adjusted to 3.5, 3.8, 3.4 and 3.4 kg DM/d for CON, CTS, RPS and LNS, respectively, to reflect decreasing animal requirements as lactation progressed (Table 1). Thus, in summer, concentrates were formulated to provide similar amounts of energy (45 ± 1.5 MJ ME/d) and crude protein (0.50 ± 0.07 kg/d), and the concentrates containing oilseeds were formulated to supply 0.37 ± 0.01 kg/d of fatty acids, compared to 0.12 kg/day of the CON concentrate. Supplementary concentrate treatments were offered individually in equal portions in the milking parlor.

From weeks 23–27 (autumn period), oilseed supplementation was suspended for all groups, and the 60 cows were managed together as a single group on the same pasture previously described and received a commercial concentrate mixture fed at 4.5 kg/d (as fed) in two equal feeds. The commercial concentrate ration (1432 Concentrados Cisternas, Osorno, Chile) supplied 140 g DM crude protein/kg and contained steam-rolled corn, ground corn, rolled barley, wheat bran, dried distillers grains with solubles, ground beans and rice bran.

The daily concentrate allocation was divided into two rations and individually offered during milking (0530 and 1600 h), and total consumption was confirmed by observation. The cows had continuous access to clean fresh water throughout the study.

### 2.2. Grazing and Feed Management and Measurements

A grazing area of around 20 ha was used for the study. This area was divided into 3 blocks to balance for soil fertility and sward age. The blocks were further subdivided into four equal paddocks, permanently fenced and randomly assigned to one of the 4 dietary treatments. The treatment groups grazed by means of strip-grazing. The electric temporary fences allowed offering cows a fresh allocation of pasture following the a.m. milking and avoided the return of the cows to the previous days grazing area. For all treatments, target post-grazing compressed residual heights were 5.5 cm to provide herbage allowances of around 20 kg DM/cow. The daily grazed area was adjusted to maintain the target post-grazing residual height.

Compressed herbage heights were measured daily before and after grazing in each paddock using a rising plate-meter (diameter 36.5 cm and 3.0 kg/2; F200 Farmworks, Feilding, New Zealand) with a minimum of 60 measurements made at random while traversing in zigzag across each grazing area.

Pre-grazing herbage mass was measured once a week in 3 rectangular framed plots (50 × 100 cm) selected at random in each paddock. In each frame, the herbage was cut with a handheld electric clipper above 3 cm, collected and weighed. Pasture density above 3 cm was calculated for each frame by dividing herbage mass by cutting depth. To determine cutting depth, 4 measurements of herbage height were made using a rising platemeter before and after cutting. A subsample of the pasture was oven dried (60 °C for 48 h) for DM determination and chemical composition analysis.

Pasture characteristics were recorded during the spring and summer periods. In the autumn period, when all cows were grazed as a single group, pasture characteristics were not recorded.

Concentrate ingredients were sampled weekly throughout the study, composited every 3 weeks, oven dried (60 °C × 48 h), and milled through a 1 mm sieve prior to chemical analysis. The dried pasture samples (from herbage mass measurement) were milled through a 1 mm sieve and composited every 2 weeks, prior to chemical composition analysis. The chemical composition of feedstuffs was conducted as previously reported [10] by methods outlined by the AOAC International [15] and included ash, crude protein (CP), EE, starch (only in the concentrates), acid detergent fiber (ADF), neutral detergent fiber (NDF), and gross energy (GE) contents.

### 2.3. Animal Measurements

Individual milk yields for all cows were recorded automatically at each milking and averaged weekly (DeLaval Alpro MM15; DeLaval International, Tumba, Sweden). Also on a weekly basis, a composite milk sample of two consecutive milkings was obtained and analyzed for milk fat, protein, lactose and urea concentrations using infrared spectroscopy (MilkoScan 4000, Foss Electric, Hillerød, Denmark). Energy corrected milk yield (ECMY) standardized to 4% fat, 3.2% crude protein and 4.8% lactose, was calculated using the equation of Tyrrell and Reid [16].

Cow body weights were recorded individually after each milking using an automatic electronic scale (DeLaval) and averaged weekly. Every 2 wk, body condition [BCS; scale: 1–5; [17]] and locomotion scores [scale: 1–3 where 1 = normal and 3 = severely lame; [18]] was assessed by two trained individuals. All records of mastitis incidence and the reproductive performance of the cows were recorded. Cows were inseminated after visual observation of estrus. Pregnancy was diagnosed via an ultrasound scan carried out by a veterinarian. First service was the first-time cows were bred and conception was the first positive scan corresponding to a service date. The fertility measurements evaluated were days from calving to first service and calving to conception, number of services per conception, 3-week submission rate defined as the percentage of cows inseminated at least once in the first 3 wk of the breeding period, conception rates to first service, second service and cumulative, defined as the percentage of cows pregnant after the first and second insemination and at the end of the breeding period, respectively, and 100-day in-calf rate defined as the percentage of cows pregnant 100 d after calving.

Individual pasture intakes were estimated on weeks 8, 19 and 27 of the study to coincide with CH_4_ measurement weeks through a process of back calculation according to Equations (1)–(4), for which energy requirement models [19,20], estimated pasture and concentrate ME contents [21] and animal data obtained in the present study, were used:

Pasture DM intake (kg/d) = pasture ME supply (MJ/d)/pasture ME content(MJ/kg DM)(1)

Pasture ME supply (MJ/d) = total ME requirement (MJ/d) − concentrate ME supply (MJ/d)(2)

Total ME requirement (MJ/d) = Sum of ME requirements for maintenance, milk production, body mass change, pregnancy + ME requirement for grazing activity allowance(3)

Concentrate ME supply (MJ/d) = concentrate DMI (kg/d) × concentrate ME content (MJ/kg)(4)

### 2.4. Methane Emissions

Individual measurements of CH_4_ emissions were carried out every 24 h for 6 consecutive days in weeks 8, 19 and 27 of the study using the sulfur hexafluoride (SF_6_) tracer gas technique [22]. Cow permeation tubes (National Institute of Water & Atmospheric Research, Wellington, New Zealand) had an initial SF_6_ charge of 2792 ± 104 mg and an estimated lifespan of around 65 weeks. Following 8 weeks of calibration by incubation at 39 °C and twice-weekly weighing, the SF_6_ release rates of permeation tubes predicted by Michaelis–Menten kinetics [23] were 4.2 ± 0.33 mg/d and ranged from 3.4 to 4.9 mg/d. The permeation tubes were dosed orally to the cows two weeks before the first CH_4_ measurement. The V-shaped PVC canisters were evacuated to at least 99 kPa vacuum before gas collection over 24-h, and flow restriction was achieved by means of a stainless-steel capillary tube cut to 30 mm length and crimpled to achieve an initial sampling rate of ~0.35 mL/min. At the end of the sampling period, vacuum within the canister was ~60 kPa and after over-pressure with ultra-high-purity N gas, reached ~20 kPa above atmospheric pressure. At least 1 h after N gas pressurization, four 30 mL sub-samples were taken from each canister using a 23-gauge needle attached to a 50 mL syringe and transferred into 4 vacuum glass vials for determination of CH_4_ and SF_6_ concentrations. During the same periods, background concentrations of CH_4_ and SF_6_ were sampled daily using 4 canisters located just outside the electric fence. Background concentrations were averaged daily to give a single estimate for all cows.

Duplicate gas samples were analyzed using gas chromatography (Perkin Elmer Clarus 600; Waltham, MA, USA). Methane separation was achieved using a Carboxen 1010 plot column (15 m × 0.32-mm ID, Supelco, Sigma-Aldrich, St. Louis, MO, USA) and detection was performed with a flame ionization detector operating at 250 °C. Gas SF_6_ separation was achieved using an Elite-GC GS Molesieve column (30 m × 0.53-mm ID × 50-μm film thickness, Perkin Elmer, Waltham, MA, USA) and detection was by electron capture detector operated at 300 °C. For each measurement period, daily CH_4_ data were averaged per cow to obtain one value per animal and season for the statistical analysis.

### 2.5. Milk and Feed Fatty Acid (FA) Content

Feed samples were freeze-dried and analyzed by a combined direct transesterification and solid-phase extraction method [24]. Milk FA determination included milk fat separation by double centrifugation [10] and FA preparation of the methyl esters (FAME) [25]. Fatty acid separation was achieved using a 100 m SP-2560 column (Supelco, Bellefonte, PA, USA) in a GC-2010 Plus Shimadzu gas chromatograph (Shimadzu, Kyoto, Japan) equipped with a flame ionization detector. The injector and detector temperature were 250 °C and the GC was operated at two complementary GC temperature programs that plateaued at 175 °C and 150 °C. In addition, a 100 m SLB-IL111 ionic liquid column (Supelco, Bellefonte, PA, USA) was used to confirm the identification of several 18 carbon biohydrogenation intermediates such as conjugated linoleic acid isomers and others. With both columns, hydrogen gas was used as carrier gas with a constant flow rate of 1 mL/min. The reference standards, individual FAME, and FA standard mixtures used for identification and recovery efficiency purposes have been previously reported [10].

### 2.6. Statistical Analyses

Because of the change in the amount and composition of concentrate supplemented, data were analyzed separately for each season spring, summer and autumn.

Pasture variables were analyzed as:

*y_ij_* = overall mean + treatment*_i_* + week*_j_* + treatment × week*_ij_* + error*_ij_*

where *y_ij_* corresponds to the pasture response *y* to the *i*th treatment (CON, LNS, RPS or CTS) in the *j*th week of each season.

Milk production and composition, body mass and body condition scores were analyzed with the following mixed model:

*y_ijk_* = overall mean + treatment*_i_* + week*_j_* + treatment*_i_* × week*_j_* + cow(random)*_k_* + error*_ijk_*

where *y_ijk_* corresponds to the response *y* of the *k*th animal to the *i*th treatment (CON, LNS, RPS or CTS) in the *j*th week of each season. In addition, initial milk production and the evaluator were included as covariables in the milk production and the body condition score models, respectively.

Data collected only once per season (CH_4_ emissions, milk FA profile, estimated pasture and total DM intake, and length of calving to first and second services and to conception) were analyzed as:

*y_ij_* = overall mean + treatment*_i_* + error*_ij_*

where *y*_ij_ corresponds to the response *y* to the *i*th treatment (CON, LNS, RPS or CTS) in each season.

Locomotion scores were analyzed by binary logistic regression, with the inclusion of the evaluator as a random co-variable:

*y_ijkl_* = overall mean + treatment_*j*_ + week_*i*_ + treatment × week_*ij*_ + cow(random)_*k*_ + evaluator(random)_*l*_ + error_*ijkl*_

where *y_ijkl_* corresponds to the locomotion scores of the *k*th cow in the *i*th treatment in the *j*th week as assessed by the *i*th evaluator.

Frequency of and number of treatments for mastitis, and categorical reproduction results, were analyzed by binary logistic regression:

*y_ij_* = overall mean + treatment_*i*_ + error_*ij*_

where *y_ij_* corresponds to the response *y* to the *j*th treatment. Significance was declared at *p* < 0.05 and tendencies at 0.05 ≤ *p* < 0.10. When treatment effect was significant means were separated using Tukey’s HSD contrasts. All statistical analyses were conducted using JMP 13.0.0 (SAS Institute Inc., Cary, NC, USA) software.

## 3. Results

### 3.1. Diet Chemical Composition and FA Profile

Reported differences in diet proximate composition and FA profile are numerical as chemical analyses were not replicated to allow for a statistical comparison (Table 1). In the spring and summer seasons, based on chemical composition and daily allowance of concentrates, the CON concentrate supplied on average 52% less EE than the oilseed concentrates. The CTS concentrate supplied more NDF and ADF than the other treatments. The content of FA differed among concentrates, with RPS having higher oleic content, CTS having a higher linoleic and palmitic content and LNS having a higher linolenic content, in both spring and summer seasons. Pasture nutrient composition varied throughout the study, and in general, the summer season had lower CP and EE and higher DM and NDF contents than the spring and autumn seasons.

### 3.2. Pasture Characteristics

In general, pasture variables were unaffected by treatments (Table 2). In spring (*p* = 0.11) and summer (*p* = 0.58), pregrazing herbage mass > 3 cm was not affected by treatments with a mean value of 3940 and 2641 kg of DM/ha, respectively. Herbage allowance did not differ between treatments, with cows offered on average 21.9 and 25.2 kg DM/d in the spring (*p* = 0.56) and summer (*p* = 0.78) seasons, respectively. Post-grazing HM above 3 cm averaged 901 kg DM/ha in spring and 980 kg DM/ha in summer and was not affected by oilseed supplementation (*p* = 0.40 y *p* = 0.75, respectively).

### 3.3. Milk Yield and Milk Composition

Compared to CON, milk yield decreased by RPS in both the spring and summer periods (both *p* < 0.05), and by CTS in the summer period (*p* < 0.05; Table 3). The ECMY was not affected by treatments in spring (*p* = 0.91), and in summer RPS decreased (*p* < 0.05) ECMY compared to CON and LNS. In the autumn (carryover period), there were no differences in milk yield (*p* = 0.18) or ECMY (*p* = 0.18) between treatments.

Milk protein concentration was not affected by treatments in spring (*p* = 0.18). In summer, CTS increased (*p* < 0.05) milk protein concentration compared to CON. Milk protein yield in spring was not affected by treatments (*p* > 0.05) and in summer, RPS decreased (*p* < 0.05) milk protein yield compared to CON. There were no differences between treatments in milk protein concentration (*p* = 0.74) or milk protein yield (*p* = 0.18) in autumn.

Compared to CON, CTS increased (*p* < 0.05) milk fat concentration in both spring and summer. Milk fat yield in spring was not affected by treatments (*p* = 0.17) and in summer, RPS decreased (*p* < 0.05) milk fat yield compared to LNS. In the autumn, there were no differences in milk fat concentration (*p* = 0.67) or milk fat yield (*p* = 0.21) between treatments.

There were no differences between treatments in milk lactose concentration in spring (*p* = 0.18), summer (*p* = 0.23) or autumn (*p* = 0.40) seasons. There were no differences between treatments in milk lactose yield in spring (*p* = 0.14) or autumn (*p* = 0.33). In summer, RPS decreased (*p* < 0.05) milk lactose yield compared to CON.

There were no differences between treatments in milk urea N concentration in spring (*p* = 0.45) or autumn (*p* = 0.68). In summer, RPS decreased (*p* < 0.05) milk urea N concentration compared to CON.

### 3.4. Milk FA Profile

The FA profile of milk from the spring, summer and autumn periods is presented in Table 4. Compared to CON, supplementation with CTS decreased most medium-chain FA (8:0, 12:0, 14:0 and 15:0; all *p* < 0.05) in spring, and in summer, 12:0 was reduced (*p* < 0.05). In spring, FA 10:0 decreased by CTS and RPS, compared to LNS (*p* < 0.05). The FA 8:0 through 16:0 were not affected by treatments in the autumn period (*p* ≥ 0.71). Palmitic acid (16:0) was not affected by treatments in spring (*p* = 0.30), but in summer it increased (*p* < 0.05) by CTS compared to CON. Stearic acid (18:0) increased (*p* < 0.05) by CTS and RPS in spring, but decreased (*p* < 0.05) by CTS and LNS in summer compared to CON. Palmitic and stearic acids were not affected by treatments in autumn (*p* ≥ 0.081). Compared to CON, oilseeds did not affect oleic acid in any period, but in spring, the content of oleic acid was greater in RPS than LNS (*p* < 0.05).

Compared to CON, CTS increased milk FA 10t-18:1, 11t-18:1 and 9c,11t-18:2 in spring and summer (all *p* < 0.05). As compared to CON, 18:2 n-6 increased by CTS (*p* < 0.05) in spring, and decreased by LNS and RPS (*p* < 0.05) in summer. Compared to CON, total conjugated linoleic acid content increased (*p* < 0.05) by LNS in spring and by CTS (*p* < 0.05) in summer. The content of 18:3 n-3 increased by LNS and decreased by CTS, compared to CON in both the spring and summer seasons, and similar results were observed for total n3 FA in spring and summer (all *p* < 0.05). In autumn, RPS had a higher total n3 FA than CTS or LNS (*p* < 0.05).

Treatments had no effect on total milk saturated FA content in either spring (*p* = 0.14), summer (*p* = 0.25) or autumn (*p* = 0.35) seasons. Milk branched chain FA decreased with CTS compared to CON in spring and summer (both *p* < 0.05), but not in autumn (*p* = 0.55). Milk monounsaturated FA (MUFA) increased (*p* < 0.05) in spring with CTS compared to LNS, but did not differ with CON, and no differences between treatments were observed in summer (*p* = 0.33) or autumn (*p* = 0.48). In spring, total polyunsaturated FA (PUFA) were higher (*p* < 0.05) in the LNS treatment than in CON or RPS treatment, and in summer, LNS and CTS had higher (*p* < 0.05) milk PUFA than RPS, but did not differ from CON. In summer, milk total highly unsaturated FA were lower with CTS than with the other treatments (*p* = 0.03), and no differences between treatments were observed in spring (*p* = 0.22) or autumn (*p* = 0.10).

### 3.5. Estimated Dry Matter Intake and Methane Production

Estimated pasture DM intake in spring (*p* = 0.97) was not affected by treatments (Table 5). In summer, compared to CON, RPS decreased pasture DM intake (*p* < 0.05) and total DM intake.

There were no differences between treatments in total CH_4_ production with a mean value of 391 g/d in spring (*p* = 0.12), 465 g/d in summer (*p* = 0.61) and 448 g/d in autumn (*p* = 0.40). In spring, the CTS treatment decreased CH_4_ emission per kg of DM intake (CH_4_ yield) and CH_4_ emission per unit of gross energy intake (CH_4_ conversion factor; Ym) compared to CON (both *p* < 0.05). These differences were no longer apparent in the summer period (*p* = 0.41). Methane emission per kg of milk (CH_4_ intensity) was not affected (*p* > 0.09) by treatments in the spring (*p* = 0.20) or summer (*p* = 0.09) periods. There were no effects of oilseed supplementation on CH_4_ production, yield or intensity in the autumn period (*p* ≥ 0.19).

### 3.6. Animal Health and Reproduction

Treatments had no effects on cow body mass or BCS in spring (*p* = 0.09 and *p* = 0.97), summer (*p* = 0.21 and *p* = 0.98) or autumn (*p* = 0.67 and *p* = 0.97), respectively (Table 6). Locomotion score (LCS) in spring was lower in the cows fed CTS than RPS (*p* < 0.05), and in all oilseed treatments compared to CON in summer (*p* < 0.05). There were no treatment effects for cow LCS in autumn (*p* = 0.11).

Cow conception rates (*p* > 0.18) and number of services to conception (*p* = 0.13) were not affected by treatments (Table 6). However, compared to CON, a lower 3-wk submission rate (*p* = 0.04) and tendency for longer interval to first service (*p* = 0.08) were observed with the LNS treatment. Clinical mastitis incidence (*p* = 0.18) and number of treatments per mastitis episode (*p* = 0.15) were not affected by treatments during the study.

## 4. Discussion

The aim of this long-term study was to evaluate, under grazing conditions, the inclusion of whole oilseeds in dairy cow diets as a CH_4_ mitigation strategy. The type of cows, diets and grazing management used in the present study are in general representative of many grazing dairy systems from temperate climates of the southern hemisphere. The diets, based on grazed grass, had a forage to concentrate ratio of around 70:30 and the CON diet (without oilseeds) had 3.8% EE in spring. The oilseed diets contained on average 6.0% and 6.1% EE content in spring and summer, respectively, which is within the recommended level of <7% fat inclusion in dairy cow diets, to avoid reduction of DM intake and fiber digestibility [8]. These levels were targeted using whole unprocessed oilseeds rather than pure oils, because we wanted to test a nutritional CH_4_ mitigation strategy that could be adopted by most local farmers, who lack the equipment and facilities to process oilseeds, and also to reduce the occurrence of milk fat depression or decreased intake or fiber digestibility [26] and avoid oil rancidification [2,27]. In general, in this study, the effects observed on milk production and composition, and on methane emissions were mild, and this is most likely related to the physical form of the oil provided (whole oilseeds) [6,9], especially for rapeseed and linseed. This is in agreement with our previous work with the same oilseeds, which were also fed unprocessed to dairy cows, but under confined conditions [10].

### 4.1. Effects on Methane

In the present study, dairy cows grazing spring pasture supplemented with CTS decreased CH_4_ yield by around 14% compared to CON. This result is consistent with CH_4_ decreases reported in previous work that evaluated concentrate feeds with whole cottonseed in dairy cows fed TMR diets (14%) [10], cottonseed with preserved forage and cereal grain diets (12 and 9%) [28,29], and preserved forage and cereal grain diets supplemented with cottonseed oil (14%) [30].

In contrast, supplementation of grazing dairy cows with whole LNS or RPS had no effect on CH_4_ production under the conditions of the present study. This finding contrasts with previous reports in which dairy cows fed mixed rations supplemented with whole cracked rapeseed [31,32] and/or crushed or extruded linseed [33,34,35] had lower CH_4_ emissions. The lack of effect on CH_4_ production when supplementing with RPS and LNS in the present study, could be explained in part due to the physical form in which the oil was provided (whole unprocessed seeds). The form of presentation of the oil can affect the extent of the response of CH_4_ emissions [5], although others have reported otherwise [36,37]. It is expected that the effects on CH_4_ emissions and milk production and composition are more pronounced with processed linseed [6] and processed rapeseed [38,39] than with unprocessed oilseeds.

Lipid supplementation and CH_4_ studies conducted with grazing dairy cows are scarce. Pinares-Patiño et al. [40] sprayed pasture of grazing steers with rapeseed oil (8.9% oil in diet DM), reporting a 22% decrease in CH_4_ yield. In dairy cows, Storlien et al. [41] reported a 12% decrease in CH_4_ emissions when grazing cows were supplemented with concentrates that included crushed rapeseed (6.3% fat in diet DM). Indeed, CH_4_ emission response when adding oils or oilseeds to dairy cow diets vary depending on the oil source [9], basal diet [35], and length of supplementation.

The CH_4_ emissions obtained in the present study were numerically on average 19% and 15% higher in summer and autumn than in spring, respectively. A numerically higher CH_4_ emission in summer may be expected because of poorer grass quality with the progression of the grazing season and lower concentrate and higher grass intake levels with the progression of lactation. The NDF concentration in summer numerically increased on average by 9% and the NDF digestibility is expected to have decreased as well. An additional aspect to consider is the transient effect of oilseeds on methane emissions with the advance of the supplementation period, although higher methane production in the summer and autumn seasons was also observed in the control group. A technical aspect that may affect the methane production measured in summer and autumn is a decline over time of the release rate of SF_6_ from the permeation tubes deployed in rumen in this long-term study [42], although permeation rates were predicted by Michaelis–Menten kinetics to account for this.

An effective CH_4_ mitigation strategy must provide effects that persist in time. Yet most CH_4_ mitigation strategies have been evaluated in short term studies, where the effects are measured after 3 or 4 weeks of treatments. Seldom have CH_4_ mitigation effects been evaluated beyond this point and there is lack of results about persistency of CH_4_ mitigation effects in the scientific literature [3]. One of the strengths of the present study is the evaluation of the supplementation with oilseeds during an extended period of time (27 weeks). In the present study, the CH_4_ mitigation effects of CTS observed in spring were no longer evident in summer (20 weeks after the beginning of oilseed supplementation). This could be due to an adaptation of the ruminal microbiota to the oil contained in the seeds, as with time, the rumen microbial community tends to adapt to changing conditions through several mechanisms (Knapp et al., 2014). In the long term, adaptation can manifest as a reversal of observed CH_4_ decrease in response to a mitigation strategy. Grainger et al. [28] reported a persistent decrease in CH_4_ emissions of up to 12 wk when supplementing dairy cows with cottonseeds. In contrast, Johnson et al. [43] reported no effects on CH_4_ emissions from calving until 305 DIM, when cows were fed a mixture of cotton and canola seeds (5.6% diet fat), with CH_4_ being measured every 3 months. Woodward et al. [44] reported decreased CH_4_ emission when supplementing grazing cows with fish and flaxseed oil in a 2-week trial, but no differences between treatments in a 12-week trial. Additionally, dairy cows fed wheat in their diets had lower CH_4_ emissions at week 4, but no differences by week 10 of the study or beyond [45]. Some authors have reported persistent decreases in CH_4_ production to week 16 with nitrate supplementation [46] and to week 12 with 3-nitrooxypropanol supplementation [47].

### 4.2. Effects on Milk Yield and Composition

Lipid supplementation has been an effective approach to increase the energy density of dairy cow diets, and can be used strategically in grass-based systems, where milk production is commonly limited by energy intake [48]. In the present study, compared to the CON cows, supplementation with RPS decreased milk yield of grazing dairy cows by around 9% in spring and 16% in summer, and CTS decreased milk yield by 11% in summer. Based on estimated ME content and allowance of the concentrates used in the study, CTS certainly supplied the lowest ME content of all concentrates in both spring and summer periods. However, this was not the case with RPS, which was estimated to supply similar levels of ME to CON and higher levels than LNS. This finding is in contrast with a review [26] and a meta-analysis [49] of the addition of fats to dairy cow diets. In a another review of 18 fat supplementation experiments performed with grazing dairy cows, Schroeder et al. [50] reported an average increase of 4.5% in milk production with fat supplementation. One possible explanation for the decrease in milk production with oilseeds we observed with some treatments in some seasons, could be that the oilseeds had a negative effect on dry matter intake or fiber digestibility [26], thus compensating the benefit of the higher energy content of the diets. Results from previous work with these same oilseeds under confined conditions indicated that cottonseed diets had lower NDF digestibility than rapeseed or linseed diets [10]. When supplementing dairy cows fed conserved forages and concentrates with cottonseeds over a 12-wk period, Grainger et al. [28] reported decreased milk yield (10%) due to refusals of the supplement and thus, lower intake, although we did not observe refusals of the concentrates.

In the present study, supplementation of CTS increased milk fat concentration in both spring and summer periods compared to CON. This was surprising, as milk fat concentration is generally decreased by fat supplementation of dairy cow diets in confined feeding systems [49] and in grazing systems, although results have been variable [50]. The oilseeds used in this study, had predominately unsaturated fatty acids with different degrees of unsaturation, therefore a decrease in milk fat concentration could have been expected due to milk fat depression [51]. The higher milk fat concentration observed with CTS compared to CON in both spring and summer can possibly be a result of an increase in the availability of preformed FA for uptake by the mammary gland due to a higher supply of exogenous FA compared to CON. Additionally, the CTS concentrate supplied a higher level of NDF than the other treatments, thus rumen NDF fermentation could increase rumen acetic and butyric production (precursors of de novo FA synthesis), and stimulate de novo FA synthesis in the mammary gland, as milk fat synthesis is limited by availability of acetate in dairy cows [52].

Milk protein concentration can be increased by contents of dietary energy and, to a lesser extent, protein [53]. In this study, milk protein concentration increased with CTS supplementation in summer by an average of 0.20 percentage units. In summer the CTS concentrate supplied a 32% numerically higher protein, but 20% lower ME contents than CON. The reduced protein yield observed in RPS during summer, was likely due to reduced total intake in this treatment.

### 4.3. Effects on Milk FA Profile

In the present study, milk FA profile changed in accordance with FA intake depending on the oil source. Milk FA composition differed the most from the CON treatment when grazing cows were supplemented with CTS in both the spring and summer periods. Supplementation with unsaturated plant oils is generally associated with decreased synthesis of de novo short- and medium-chain FA in the mammary gland and a proportional increase in 18C FA [54]. These results were only observed with the CTS treatment.

Supplementation with sources of unsaturated FA generally results in milk with greater concentration of unsaturated fatty acids [55,56]. Yet, compared to CON in the present study, the milk PUFA content was only increased with LNS in the spring. This could be the result, of a moderate effect of the oil supplementation because of the physical form of the oil offered in the present study. The lack of effects observed in summer can also be related to forage maturation and higher saturated FA intake provided by grazed forages as the season progressed.

Supplementing dairy cow diets with lipids has been performed to modify milk FA profile increasing the proportion of unsaturated FA. Saturated FA have been perceived as being unhealthy when consumed in excess, whereas others, such as oleic, vaccenic, rumenic, linoleic and linolenic, are perceived as healthy for human health [57,58], associated with reports of specific fatty acids, such as omega-3 FA, having anti-inflammatory and cardiovascular protective effects [59] and conjugated cis and trans isomers of linoleic acid having anti-carcinogenic properties [26]. In the present study, oilseeds had no effect on total saturated FA concentration, but supplementation of CTS increased vaccenic, linoleic and rumenic FA compared to CON. Supplementation of LNS increased healthy FA alfa-linolenic in both spring and summer periods, and RPS increased stearic acid in spring and decreased healthy linoleic acid in summer, both compared to CON. The perception and classification of FA as “unhealthy” is being challenged with new reviews and meta-analyses of past studies that indicate that trans-FA of natural origin and saturated FA are not harmful to human health [58,60,61,62].

### 4.4. Effects on Health and Reproduction

This long-term study also evaluated the effects of treatments on variables related to dairy cow health and reproduction. Importantly, in summer all oilseeds decreased lameness score in the cows compared to CON. Previous studies indicate that changes in diet lipid content can influence the composition of the fat pad and integrity of the hoof horn [63]. However, LNS-supplemented exhibited somewhat poorer reproductive performance than CON cows, particularly at the beginning of the breeding period. This is in contrast with a recent review that reported beneficial effects of n-3 FA from flaxseed on the reproductive efficiency of dairy cattle [64]. Although we acknowledge that the experimental numbers of this trial are too small to accurately assess reproduction and health variables, we think an important contribution of this study is to offer a more holistic response to dietary methane mitigation interventions such as oilseed supplementation including these aspects. Another aspect to consider is the higher feeding costs of oilseeds dietary inclusion which would need to be offset by improved performance, as methane mitigation interventions should be economically viable to be more widely adopted by farmers.

### 4.5. Carry over Effect of Lipid Supplementation

We hypothesized that there would be carry over effects after lipid supplementation ended. However, in autumn there were no carry over effects of treatments on CH_4_ emission, milk production or FA profile. This result could not have been foreseen prior to conducting the experiment. Given that the effects on CH_4_ were not present in summer, no carry over effects in autumn would be expected. Thus, this question remains unanswered and further research withdrawing oil supplementation while it is still effective is required to address it. The effectiveness of oil supplementation is influenced by the form of presentation of the oil, the lack of physical disruption of the oilseeds, oil concentration in the oilseeds, fatty acid composition and the amount of oilseeds fed. These aspects will need consideration in future studies, including dose response studies when supplementing with whole oilseeds.

## 5. Conclusions

In the present study, whole cottonseeds fed to grazing dairy cows decreased CH_4_ yield and sustained milk production for a period of up to at least 8 weeks, but the effects did not persist after 19 weeks of supplementation, and cottonseeds improved milk FA profile by increasing stearic, linoleic and rumenic FA concentrations. In the long term, cottonseed was more effective in terms of modifying CH_4_ yield, milk production and milk fat composition than whole linseed or rapeseed. However, the results observed were mild, most likely related to the physical form of the oil (unprocessed oilseeds), and higher levels of whole oilseed supplementation may be needed to observe larger effects. There were no carryover effects of treatments, after oilseed supplementation ended. Long term studies conducted under grazing conditions are important to provide a comprehensive overview of how proposed nutritional CH_4_ mitigation strategies affect productivity, sustainability and consumer health aspects.

## Figures and Tables

**Table 1:** Concentrate ingredient and chemical composition and major fatty acids (FA) content in concentrates and pasture in spring, summer and autumn seasons.

	Concentrates ^1^ in SPR ^2,3^				Concentrates ^1^ in SMR ^2,3^				Pasture^3^		
	CON	CTS	RPS	LNS	CON	CTS	RPS	LNS	SPR ^2^	SMR ^2^	AUT ^2^
Ingredient composition on DM basis, %											
Steam-flaked corn	83	48	63	67	79	47	59	66	-	-	-
Whole rapeseed	-	-	20	-	-	-	21	-	-	-	-
Whole cottonseed	-	48	-	-	-	47	-	-	-	-	-
Whole linseed	-	-	-	20	-	-	-	20	-	-	-
Rapeseed cake	14	-	13	8	15	-	13	8	-	-	-
Vitamin and mineral mixture ^4^	3.8	4.1	4.8	4.7	5.6	5.3	5.9	6.0	-	-	-
Chemical composition											
DM, g/kg fresh matter	895	904	903	905	917	920	924	926	161	215	167
Ash, g/kg DM	16	26	20	18	18	27	21	19	114	118	114
CP, g/kg DM	100	143	122	111	103	143	121	112	210	177	192
ME, MJ/kg	13.3	11.2	13.1	13.1	13.2	11.1	12.9	12.9	11.5	10.6	-
NDF, g/kg DM	98	225	131	116	104	246	135	121	459	500	474
ADF, g/kg DM	43	154	81	62	40	169	76	58	270	289	275
EE, g/kg DM	53	138	135	123	47	119	132	121	33	31	33
Starch, g/kg DM	583	337	444	475	549	317	419	448	-	-	-
FA profile, g/100 g of total FA											
16:00	11.5	16.9	9.7	10.4	11.9	18.1	10.0	10.9	18.2	24.6	17.5
18:00	2.0	2.2	1.9	2.3	2.0	2.2	1.9	2.3	2.1	4.3	2.4
9c-18:1/15t-18:1/19:0ai	34.3	22.7	41.0	30.0	33.0	22.7	40.0	29.1	2.5	2.9	1.8
18:2n-6	46.6	54.4	40.3	41.2	46.5	52.4	39.8	41.5	13.6	14.3	13.5
18:3n-3	2.8	1.0	4.1	13.5	3.7	1.3	5.3	13.5	52.0	41.1	53.4

^1^ Concentrate supplement without oilseed (control; CON), with the inclusion of whole unprocessed rapeseed (RPS), whole unprocessed cottonseed with lint (CTS) and whole unprocessed linseed (LNS). ^2^ Spring period (SPR) from wk 1-16 of the study and summer period (SMR) from wk 17–22 of the study. ^3^ Values were calculated based on the chemical composition of the concentrates in weeks 8 and 19 of the study (methane measurement weeks). ^4^ Containing per kg: Ca 120 g, P 40 g, Na 60 g, Mg 35 g, Cl 90 g, S 30 g, Zn 1575 mg, Mn 2135 mg, Cu 288 mg, I 50 mg, Co 50 mg, Se 31 mg, vitamin A 101,000 IU, vitamin D 50,000 IU, vitamin E 1200 IU, biotin 71 mg, acid buf 100 g (Vetersal Rio Bueno, Veterquímica, Osorno, Chile). “-” Not included in ingredient composition; not determined in chemical composition; not detected in FA profile.

**Table 2:** Effect of oilseed supplementation on pasture characteristics in the spring and summer seasons.

	Spring ^1,2^						Summer ^1,2^					
Item	CON	CTS	RPS	LNS	SEM	*p* Value	CON	CTS	RPS	LNS	SEM	*p* Value
Pregrazing herbage height, cm	14.6	12.7	13.5	14.1	0.57	0.06	8.3	8.9	10.0	8.0	0.76	0.31
Pregrazing herbage mass, kg of DM/ha	4276	3826	3642	4146	229	0.11	2152	2702	2698	2184	362	0.58
Herbage allowance, kg of DM/cow per d	23.0	23.4	21.2	23.0	1.30	0.56	23.6	26.7	23.0	24.6	2.70	0.78
Postgrazing herbage height, cm	5.6	5.4	5.4	5.4	0.12	0.33	5.1	5.0	5.5	5.0	0.25	0.50
Postgrazing herbage mass, kg of DM/ha	1001	961	859	924	69.0	0.40	843	986	1002	885	120	0.75
Herbage removed, kg of DM/cow per d	17.1	16.9	15.5	17.2	0.88	0.39	13.6	16.1	13.8	13.9	2.13	0.83

^1^ Concentrate supplement without oilseed (control; CON), with the inclusion of whole unprocessed rapeseed (RPS), whole unprocessed cottonseed with lint (CTS) and whole unprocessed linseed (LNS). ^2^ Spring period from wk 1–16 of the study and summer period from wk 17–22 of the study.

**Table 3:** Effects of oilseed supplementation on milk and milk component yield, milk composition throughout the study.

	Spring ^1,2^						Summer ^1,2^						Autumn ^1,2^					
Item	CON	CTS	RPS	LNS	SEM	*p* Value	CON	CTS	RPS	LNS	SEM	*p* Value	CON	CTS	RPS	LNS	SEM	*p* Value
Milk yield, kg/day	27.2 ^a^	25.7 ^a,b^	24.9 ^b^	26.6 ^a,b^	0.52	0.02	20.8 ^a^	18.6 ^b,c^	17.4 c	20.3 ^a,b^	0.51	<0.001	17.2	16.8	15.5	17.4	0.66	0.18
ECMY ^3^, kg/d	23.4	24.0	22.6	23.7	0.59	0.91	19.4 ^a^	18.5 ^a,b^	17.0 ^b^	19.7 ^a^	0.52	<0.01	17.6	18.2	17.3	18.6	0.74	0.61
Milk protein, g/kg	34.5	35.6	34.4	34.0	0.55	0.18	34.5 ^b^	36.5 ^a^	34.8 ^a,b^	34.8 ^a,b^	0.45	0.01	37.9	38.3	37.6	37.3	0.70	0.74
Milk protein yield, kg/d	0.92	0.90	0.85	0.89	0.022	0.10	0.71 ^a^	0.68 ^a^	0.61 ^b^	0.71 ^a^	0.018	<0.001	0.64	0.64	0.58	0.65	0.023	0.18
Milk fat, g/kg	28.2 ^b^	33.3 ^a^	31.3 ^a,b^	31.2 ^a,b^	1.04	0.01	33.8 ^b^	37.3 ^a^	36.5 ^a,b^	36.5 ^a,b^	0.94	0.05	42.0	44.1	42.6	42.8	1.23	0.67
Milk fat yield, kg/d	0.75	0.83	0.76	0.81	0.030	0.17	0.70 ^a,b^	0.69 ^a,b^	0.63 ^b^	0.74 ^a^	0.028	0.025	0.71	0.73	0.66	0.74	0.028	0.21
Milk lactose, g/kg	49.2	49.7	50.1	49.6	0.30	0.18	47.6	47.8	48.4	48.0	0.28	0.23	47.5	47.6	46.7	47.3	0.39	0.40
Milk lactose yield, kg/d	1.33	1.26	1.23	1.30	0.030	0.14	0.99 ^a^	0.89 ^a,b^	0.85 ^b^	0.97 ^a^	0.039	<0.001	0.81	0.80	0.73	0.83	0.034	0.22
Milk urea N, mg/dL	23.1	23.3	21.9	22.3	0.70	0.45	31.1 ^a^	30.8 ^a^	28.4 ^b^	30.3 ^a,b^	0.59	0.01	35.2	34.5	34.4	34.0	0.69	0.68
Milk Log SCC ^4^	1.74	1.68	1.89	1.62	0.101	0.25	1.98	1.86	2.08	1.89	0.088	0.27	2.17	2.01	2.26	2.07	0.095	0.25

^1^ Concentrate supplement without oilseed (control; CON), with the inclusion of whole unprocessed rapeseed (RPS), whole unprocessed cottonseed with lint (CTS) and whole unprocessed linseed (LNS). ^2^ Spring period from wk 1 to 16 of the study, summer period from wk 17 to 22 of the study and autumn period from wk 23 to 27 of the study. ^3^ ECMY = energy corrected milk yield. ^4^ SCC = somatic cell count. ^a–c^ = for each season means with different letters within rows are statistically different (*p* < 0.05).

**Table 4:** Effect of oilseed supplementation on milk fatty acid (FA) composition of lactating dairy cows on pasture in spring and summer.

	SPRING ^1,2^						SUMMER ^1,2^						AUTUMN ^1,2^					
	CON	CTS	RPS	LNS	SEM	*p* Value	CON	CTS	RPS	LNS	SEM	*p* Value	CON	CTS	RPS	LNS	SEM	*p* Value
FA, g/100 g of total FA ^3,4^																		
8:0	1.13 ^a^	0.69 ^b^	1.01 ^a^	0.85 ^a,b^	0.083	<0.01	0.87	0.89	0.94	1.11	0.067	0.06	1.06	0.99	1.05	1.08	0.071	0.83
10:0	3.34 ^a,b^	2.04 ^b^	2.85 ^b^	3.28 ^a^	0.140	<0.001	2.64	2.25	2.63	2.83	0.146	0.05	2.88	2.74	2.93	3.05	0.132	0.43
12:0	3.73 ^a^	2.29 ^b^	3.20 ^a^	3.74 ^a^	0.148	<0.001	3.04 ^a^	2.49 ^b^	3.17 ^a^	3.07 ^a^	0.118	<0.01	3.22	3.10	3.29	3.43	0.144	0.44
14:0	11.46 ^a,b^	8.49 ^c^	10.45 ^b^	11.87 ^a^	0.324	<0.001	10.61	9.88	10.84	10.74	0.263	0.05	10.68	10.35	10.30	11.04	0.281	0.23
15:0	1.07 ^a,b^	0.88 ^c^	0.97 ^b,c^	1.19 ^a^	0.042	<0.001	1.03 ^a,b^	1.01 ^b^	1.12 ^a^	1.04 ^a,b^	0.029	0.04	0.98	1.01	0.99	1.01	0.020	0.69
16:0	24.57	24.55	23.03	25.11	0.789	0.30	24.80 ^b^	28.01 ^a^	26.59 ^a,b^	26.54 ^a,b^	0.713	0.02	24.01	24.65	23.84	25.30	0.517	0.19
Total 16:1 cis	1.89 ^a^	1.48 ^b^	1.83 ^a^	1.77 ^a^	0.067	<0.01	1.89	1.95	1.90	1.97	0.069	0.82	2.10	2.02	1.96	2.13	0.063	0.22
18:0	11.80 ^b^	14.97 ^a^	13.80 ^a^	11.23 ^b^	0.440	<0.001	12.72 ^a^	11.04 ^b^	12.19 ^a,b^	10.94 ^b^	0.393	<0.01	11.07	11.65	12.08	11.03	0.328	0.081
10t-18:1	0.41 ^b^	0.63 ^a^	0.40 ^b^	0.41 ^b^	0.030	<.0001	0.38 ^b^	0.56 ^a^	0.32 ^c^	0.31 ^c^	0.015	<0.001	0.39	0.37	0.39	0.38	0.011	0.34
11t-18:1	1.72 ^b^	2.50 ^a^	1.71 ^b^	2.14 ^a,b^	0.120	<0.001	1.64 ^b^	2.04 ^a^	1.75 ^a,b^	1.59 ^b^	0.086	<0.01	1.97	1.90	1.98	1.94	0.089	0.93
9c-18:1	23.09 ^a,b^	23.81 ^a,b^	25.35 ^a^	21.66 ^b^	0.825	0.03	25.49	24.30	23.95	23.95	0.747	0.42	25.07	25.22	24.71	23.57	0.688	0.33
18:2 n-6	1.59 ^b^	2.08 ^a^	1.49 ^b^	1.45 ^b^	0.061	<0.001	1.50 ^a^	1.56 ^a^	1.19 ^b^	1.28 ^b^	0.041	<0.001	1.67	1.56	1.72	1.51	0.060	0.06
9c,11t-18:2	0.87 ^b^	0.98 ^a^	0.81 ^b^	1.09 ^a,b^	0.067	0.03	0.84 ^b^	1.11 ^a^	0.88 ^b^	0.94 ^a,b^	0.053	<0.01	1.16	1.05	1.05	1.08	0.060	0.56
18:3 n-3	0.80 ^b^	0.58 ^c^	0.87 ^b^	1.15 ^a^	0.027	<0.001	0.76 ^b^	0.67 ^c^	0.71 ^b,c^	0.93 ^a^	0.025	<0.001	0.99	0.92	1.00	0.91	0.029	0.06
Total SFA ^5^	60.82	57.90	58.93	60.82	1.067	0.14	59.41	58.71	61.07	59.66	0.848	0.25	57.33	57.88	57.90	59.34	0.812	0.35
Total BCFA ^6^	2.40 ^a^	2.03 ^b^	2.33 ^a^	2.23 ^a,b^	0.066	<0.01	2.36 ^a^	2.08 ^b^	2.23 ^a,b^	2.16 ^a,b^	0.065	0.02	2.21	2.15	2.21	2.14	0.045	0.55
Total MUFA ^7^	32.82 ^a,b^	36.15 ^a^	35.00 ^a,b^	32.17 ^b^	1.048	0.03	34.76	34.92	33.15	33.67	0.797	0.33	35.46	35.30	35.03	33.96	0.732	0.48
Total PUFA ^8^	3.93 ^b^	4.19 ^a,b^	3.86 ^b^	4.52 ^a^	0.131	<0.01	3.76 ^a,b^	3.95 ^a^	3.45 ^b^	3.92 ^a^	0.097	<0.01	4.59	4.28	4.59	4.24	0.120	0.07
Total HUFA ^9^	0.41	0.36	0.42	0.41	0.021	0.22	0.43 ^a^	0.35 ^b^	0.40 ^a^	0.40 ^a^	0.018	0.03	0.46	0.46	0.50	0.43	0.019	0.10
Total n6	1.76 ^b^	2.27 ^a^	1.66 ^b^	1.59 ^b^	0.065	<0.001	1.67 ^a^	1.75 ^a^	1.35 ^b^	1.42 ^b^	0.043	<0.001	1.85	1.74	1.90	1.66	0.064	0.05
Total n3	1.06 ^b^	0.77 ^c^	1.15 ^b^	1.44 ^a^	0.032	<0.001	1.04 ^b^	0.86 ^c^	0.97 ^b^	1.21 ^a^	0.029	<0.001	1.30 ^a,b^	1.22 ^b^	1.35 ^a^	1.21 ^b^	0.033	0.01
Total CLA ^10^	1.04 ^b^	1.10 ^a,b^	0.97 ^b^	1.33 ^a^	0.072	0.01	1.00 ^b^	1.28 ^a^	1.05 ^b^	1.16 ^a,b^	0.055	<0.01	1.36	1.24	1.26	1.29	0.062	0.54
Unknown FA	1.25 ^a^	0.77 ^b^	1.06 ^a,b^	0.86 ^b^	0.085	<0.01	0.91	1.01	1.17	1.30	0.144	0.26	1.22	1.22	1.14	1.09	0.113	0.80

^1^ Concentrate supplement without oilseed (control; CON), with the inclusion of whole unprocessed rapeseed (RPS), whole unprocessed cottonseed with lint (CTS) and whole unprocessed linseed (LNS). ^2^ Spring period from wk 1–16 of the study, summer period from wk 17–22 of the study and autumn period from wk 23–27 of the study. ^3^ FA = fatty acids. ^4^ Values are for methane measurement in weeks 8 (spring) and 19 (summer) of the study. ^5^ SFA = saturated fatty acids. ^6^ BCFA = branched chain fatty acids. ^7^ MUFA = monounsaturated fatty acids. ^8^ PUFA = polyunsaturated fatty acids. ^9^ HUFA = highly unsaturated fatty acids (≥ 20 carbons and ≥ 3 double bonds). ^10^ CLA = conjugated linoleic acid. ^a–c^ = for each season means with different letters within rows are statistically different (*p* < 0.05).

**Table 5:** Effect of oilseed supplementation on dry matter intake and methane (CH_4_) production of lactating dairy cows on pasture.

	Spring ^1,2^						Summer ^1,2^						Autumn ^1,2^					
	CON	CTS	RPS	LNS	SEM	*p* Value	CON	CTS	RPS	LNS	SEM	*p* Value	CON	CTS	RPS	LNS	SE	*p* Value
Dry matter intake, kg/day																		
Concentrate	5.1	5.2	4.6	4.8	-	-	3.5	3.8	3.4	3.4	-	-	4.0	4.0	4.0	4.0	-	-
Grass intake ^3^	12.6	12.9	12.6	12.6	0.51	0.97	14.5 ^a^	14.1 ^a,b^	12.8 ^b^	13.4 ^a,b^	0.42	0.04	13.8	13.5	13.2	13.4	0.48	0.85
Total intake	18.0	18.3	17.3	17.6	0.51	0.46	18.0 ^a^	17.9 ^a^	16.2 ^b^	16.7 ^a,b^	0.42	<0.01	17.8	17.5	17.2	17.4	0.48	0.85
Methane																		
CH_4_, g/day	412	359	393	401	16.1	0.12	469	484	455	454	17.7	0.61	450	432	439	472	17.4	0.40
CH_4_, g/kg DMI ^4^	22.7 ^a^	19.5 ^b^	23.0 ^a^	22.9 ^a^	0.78	0.01	26.2	27.3	28.5	27.3	0.97	0.41	24.6	25.0	26.0	27.2	0.90	0.19
CH_4_, g/kg milk yield	16.4	15.4	17.7	15.5	0.87	0.20	22.5	25.0	25.0	21.8	1.08	0.09	28.0	26.5	29.5	27.9	1.92	0.77
Ym, % ^5^	7.2 ^a^	6.2 ^b^	7.3 ^a^	7.3 ^a^	0.25	<0.01	8.3	8.6	9.0	8.6	0.31	0.41	7.8	7.9	8.3	8.6	0.29	0.19

^1^ Concentrate supplement without oilseed (control; CON), with the inclusion of whole unprocessed rapeseed (RPS), whole unprocessed cottonseed with lint (CTS) and whole unprocessed linseed (LNS). ^2^ Spring period from wk 1 to 16 of the study, summer period from wk 17 to 22 of the study and autumn period from wk 23 to 27 of the study. ^3^ Estimated individually using energy algorithms [13,14] based on body mass, body mass change, milk production, milk composition, gestation data and estimated feed ME content [15]. ^4^ DMI = dry matter intake. ^5^ Ym = methane conversion factor (% of gross energy intake). ^a,b^ = for each season means with different letters within rows are statistically different (*p* < 0.05).

**Table 6:** Effect of oilseed supplementation on reproductive and health performance of grazing dairy cows.

	Treatments ^1^					
Item	CON	CTS	RPS	LNS	SEM	*p* Value
3-week submission rate, %	20.0 ^a^	13.3 ^a,b^	33.3 ^a^	0.0 ^b^	-	0.042
100-day in-calf rate, %	53.3	33.3	46.7	33.3	-	0.60
Conception rate to first service, %	66.7	66.7	46.2	85.7	-	0.18
Conception rate to second service, %	86.7	93.3	92.3	92.9	-	0.92
Cumulative conception rate, %	93.3	93.3	73.3	80.0	-	0.31
Calving to first service interval, days	88.6	97.2	80.2	102.4	6.23	0.08
Calving to conception interval, days	98.6	110.4	100.8	105.8	7.85	0.70
N° of services to conception, weighted means	1.32	1.30	1.52	1.08	-	0.13
Clinical mastitis, %	6.7	0.0	20.0	13.3	-	0.18
N° treatments per mastitis, weighted means	1.00	-	2.00	1.00	-	0.15
Spring ^2^						
Body mass, kg	532	541	532	535	2.62	0.09
Body condition score	3.12	3.10	3.10	3.06	0.083	0.97
Locomotion score, weighted means	1.51 ^a,b^	1.34 ^b^	1.59 ^a^	1.44 ^a,b^	0.044	<0.01
Summer ^2^						
Body mass, kg	551	562	553	552	4.40	0.21
Body condition score	3.11	3.07	3.08	3.12	0.089	0.98
Locomotion score, weighted means	1.7 ^a^	1.5 ^b^	1.5 ^b^	1.5 ^b^	0.041	0.04
Autumn ^2^						
Body mass, kg	555	564	562	560	5.52	0.67
Body condition score	3.09	3.03	3.09	3.07	0.097	0.97
Locomotion score, weighted means	1.7	1.5	1.5	1.4	0.079	0.11

^1^ Concentrate supplement without oilseed (control; CON), with the inclusion of whole unprocessed rapeseed (RPS), whole unprocessed cottonseed with lint (CTS) and whole unprocessed linseed (LNS). ^2^ Spring period from wk 1 to 16 of the study, summer period from wk 17 to 22 of the study and autumn period from wk 23 to 27 of the study. ^a,b^ = for each season means with different letters within rows are statistically different (*p* < 0.05).

## Data Availability

Not applicable.
